# Systematic Review of Interleukin-35 in Endothelial Dysfunction: A New Target for Therapeutic Intervention

**DOI:** 10.1155/mi/2003124

**Published:** 2025-02-12

**Authors:** Kai Li, Jie Feng, Meng Li, Leilei Han, Yanqing Wu

**Affiliations:** Department of Cardiology, The Second Affiliated Hospital, Jiangxi Medical College, Nanchang University, No. 1 Minde Road, Nanchang 330006, Jiangxi, China

**Keywords:** angiogenesis, endothelial dysfunction, inflammation, interleukin-35

## Abstract

Endothelial dysfunction is a significant factor in the pathogenesis of various diseases. In pathological states, endothelial cells (ECs) undergo activation, resulting in dysfunction characterized by the stimulation of inflammatory responses, oxidative stress, cell proliferation, blood coagulation, and vascular adhesions. Interleukin-35 (IL-35), a novel member of the IL-12 family, is primarily secreted by regulatory T cells (Tregs) and regulatory B cells (Bregs). The role of IL-35 in immunomodulation, antioxidative stress, resistance to apoptosis, control of EC activation, adhesion, and angiogenesis in ECs remains incompletely understood, as the specific mechanisms of IL-35 action and its regulation have yet to be fully elucidated. Therefore, this systematic review aims to comprehensively investigate the impact of IL-35 on ECs and their physiological roles in a range of conditions, including cardiovascular diseases, tumors, sepsis, and rheumatoid arthritis (RA), with the objective of elucidating the potential of IL-35 as a therapeutic target for these ailments.

## 1. Introduction

Endothelial cells (ECs), which comprise the inner lining of vessels, fulfill various roles in the body [1]. Activation of ECs can be triggered by various factors, such as hyperlipidemia, hyperglycemia, and inflammation [2]. Upon activation, ECs upregulate the expression of adhesion molecules like ICAM-1 and VCAM-1, as well as inflammatory mediators, including IL-6, IL-1*β*, and TNF-*α*^3^. Prolonged endothelial activation ultimately culminates in endothelial dysfunction [3]. Endothelial dysfunction is characterized by pathological manifestations, including inflammation [4], oxidative stress [5], pathological proliferation [6], coagulation [7], and vascular adhesion [8]. This dysfunction plays a crucial role in the pathogenesis and advancement of numerous diseases, including cardiovascular diseases [9], diabetes [10], rheumatoid arthritis (RA) [11], sepsis [12], and tumors [13].

Interleukin-35 (IL-35), a recently discovered cytokine belonging to the IL-12 family, is mainly produced by regulatory T cells (Tregs) and regulatory B cells (Bregs) [14, 15]. In ECs, IL-35 plays a multifaceted role encompassing immunomodulation, antioxidative stress, antiapoptosis, and regulation of EC activation, adhesion, and angiogenesis [6, 16–19]. Nevertheless, the precise mechanisms underlying the actions of IL-35 in ECs remain incompletely elucidated. The primary objective of this article is to provide a systematic review of the impact of IL-35 on ECs and its role in various diseases, such as cardiovascular diseases, tumors, sepsis, RA, and other conditions, with the aim of investigating its potential as a therapeutic target for these ailments.

## 2. Methods

### 2.1. Search Strategy

In June 2023, a comprehensive search was performed in PubMed, Ovid's version of MEDLINE, and EMBASE databases. The following terms were used: “Interleukin-35” OR “IL-35”, “B cell” OR “T cell” OR “macrophage”, “Endothelial cells” OR ”Endothelial function” OR “Endothelial dysfunction,” “Inflammation” OR “Angiogenesis” OR “Cell adhesion” OR “Atherosclerosis” OR “Cardiovascular diseases” OR “Sepsis” OR “Rheumatoid arthritis” OR “Tumors”, combined with AND, such as (“Endothelial function” OR “Endothelial dysfunction”) AND (“IL-35” OR “Interleukin-35”). This search primarily focused on basic research studies, including in vitro and animal studies, with a focus on IL-35's molecular mechanisms in ECs. Clinical studies were included for reference to demonstrate IL-35's relevance in endothelial dysfunction-related diseases. The search was limited to studies from January 2000 to present, excluding reviews, clinical trials, and without explicit investigation into IL-35. The review followed PRISMA guidelines for systematic reviews.

### 2.2. Study Selection

The study selection process aimed to identify all relevant published studies on IL-35, focusing on the following key areas: (1) structure and origin of IL-35, (2) downstream signaling pathways and biological functions of IL-35, and (3) effects of IL-35 on ECs and endothelial function in various diseases. While providing a comprehensive review of IL-35's signaling pathways and biological functions, we included a more concise discussion to maintain focus on the primary research objectives. Additionally, clinical studies related to IL-35 in certain diseases were included in this review but not discussed in detail, only highlighting the relevance of IL-35 to clinical diseases. Inclusion was based on established relevance and rigor, ensuring the studies contribute to a comprehensive understanding of IL-35's role in endothelial dysfunction and related diseases.

### 2.3. Data Extraction

The titles and abstracts of all identified records from each database search were imported into the commercial reference management software EndNote (Clarivate Analytics). Duplicate records were identified and removed through manual screening. Two investigators (Kai Li and Jie Feng) independently reviewed the final list of studies for inclusion. For studies discussing IL-35's structure and function, data extraction was performed by Kai Li and Leilei Han. For studies investigating IL-35's role in endothelial function in various diseases, data extraction was performed by Kai Li and Meng Li. Any disagreements were resolved through discussion, and quality assessment tools were used as appropriate. Finally, Kai Li completed the preparation of the tables and figures, with the figures created using the online tool Figdraw.

## 3. Results

**Figure 1:**
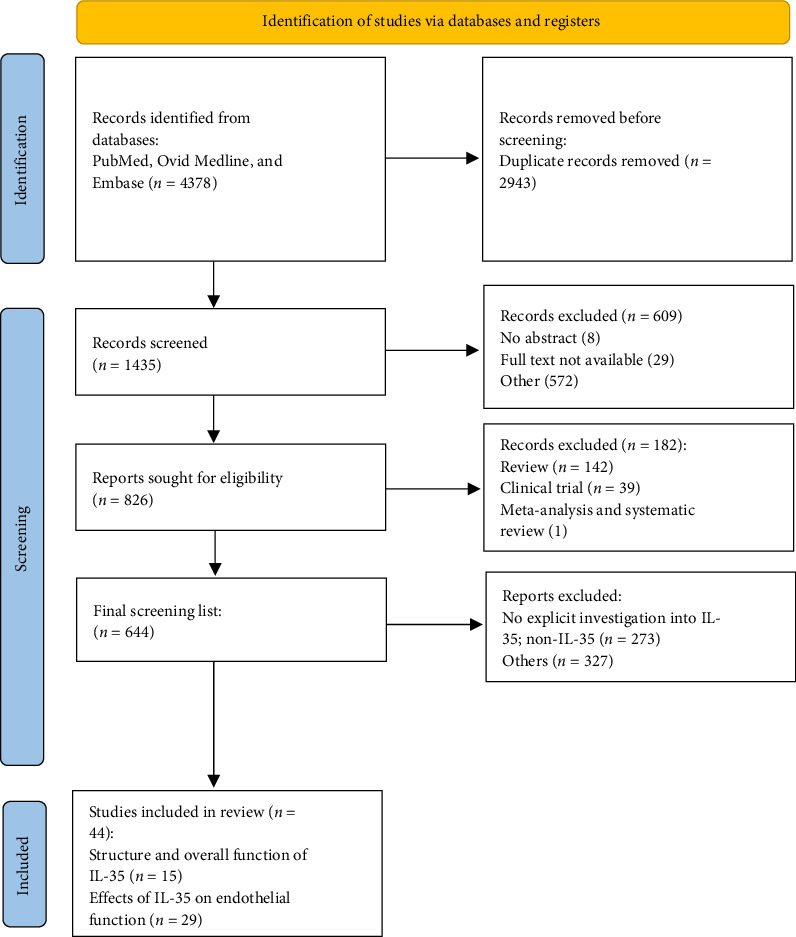
PRISMA 2020 flow diagram for new systematic reviews, which included searchers of databases and registers only. From [M. J. Page, J. E. McKenzie, P. M. Bossuyt, I. Boutron, T. C. Hoffmann, C. D. Mulrow, et al. The PRISMA 2020 statement: an updated guideline for reporting systematic reviews. BMJ 2021; 372: n71. doi: 10.1136/bmj. n71].

A total of 644 citations were reviewed, and 44 studies met the eligibility criteria and were included in the final systematic review. Among these, 15 studies focused on the structure and overall function of IL-35, while 29 studies examined the effects of IL-35 on endothelial function. The full PRISMA flow diagram is presented in Figure 1. Detailed information on studies investigating the effects of IL-35 on endothelial function in vascular diseases, tumors, and RA is provided in Tables 1–3 of the corresponding chapters, along with a comprehensive illustration of the results and relevant references. Additionally, other diseases, such as sepsis and osteoarthritis (OA), will be discussed in detail.

### 3.1. ECs and Endothelial Dysfunction

ECs, which comprise the inner lining of arteries, veins, and capillaries, fulfill a multifaceted role in the body [1]. In addition to acting as a protective barrier, these cells are involved in various physiological processes such as nutrient transport [28], coagulation and anticoagulation balance [29], regulation of vascular tone [30], expression of innate immune receptors [30], endocrine function [31], maintenance of endothelial integrity [32], control of leukocyte extravasation [33], angiogenesis [5], and overall maintenance of multiorgan health and homeostasis [34]. ECs exhibit both resting and activated states. During periods of rest, ECs regulate the secretion of vasoconstrictive and vasodilatory factors, including nitric oxide (NO) [35], prostacyclin [36], and endothelin [37], in order to uphold vascular tone, blood pressure, and blood flow. Additionally, in the resting state, ECs prevent leukocyte interaction [38] and suppress the expression of adhesion molecules like E-selectin, VCAM-1, and ICAM-1 [39]. Exposure to factors such as hyperlipidemia, hyperglycemia, and inflammation disrupts these protective mechanisms of ECs, resulting in their activation and compromising their structural integrity [2]. ECs in an activated state exhibit heightened secretion of adhesion molecules, including ICAM-1 and VCAM-1, as well as inflammatory factors such as IL-6, IL-1*β*, and TNF-*α*. These molecular changes facilitate the trans-endothelial migration of various immune cells, including monocytes [33], macrophages [40], dendritic cells [41], leukocytes [42], B-cells [43], T-cells [44], and NK-cells [45]. Prolonged endothelial activation ultimately leads to endothelial dysfunction [3]. Endothelial dysfunction is characterized by an imbalance between vasodilatory factors and constrictive factors, resulting in a predominantly provasoconstrictive state. This imbalance contributes to pathophysiological changes, including inflammation [4], oxidative stress [5], pathological proliferation [6], coagulation [7], and vascular adhesion [8]. Endothelial dysfunction is a crucial factor in the development and progression of various diseases, including cardiovascular diseases [9], metabolic syndrome [10], systemic inflammatory disease [11], sepsis [12], and tumors [13].

### 3.2. Overview of IL-35

In 1997, Devergne, Birkenbach, and Kieff [46] identified a novel dimer formed by the EBI3 and p35 subunits of IL-12, demonstrating mutual promotion of secretion between these subunits. Subsequently, Collison et al. [14] in 2007 designated this dimer as IL-35, a constituent of the IL-12 cytokine family characterized by the assembly of distinct *α*- and *β*-chains into heterodimeric proteins. The IL-12 family comprises IL-12, IL-23, IL-27, IL-35, and IL-39, each composed of specific subunits. These subunits include p35 and p40 for IL-12, p19 and p40 for IL-23, p28 and EBI3 for IL-27, p35 and EBI3 for IL-35, and p19 and EBI3 for IL-39 [47–50]. Additionally, each member of the IL-12 family possesses a corresponding receptor composed of two subunits. Specifically, the receptor subunit for IL-12 is IL-12R*β*1:IL-12R*β*2, for IL-23 is IL-23R:IL-12R*β*1, for IL-27 is IL-27R*α*:gp130, and for IL-39 is IL-23R:gp130. The receptors for IL-35 exhibit diverse and cell-specific forms, including IL-12R*β*2:IL-12R*β*2, IL-12R*β*2:gp130, gp130:gp130, and IL-12R*β*2:IL-27R*α*, which are reported to be promoted by IL-35 [8, 51, 52].

**Figure 2:**
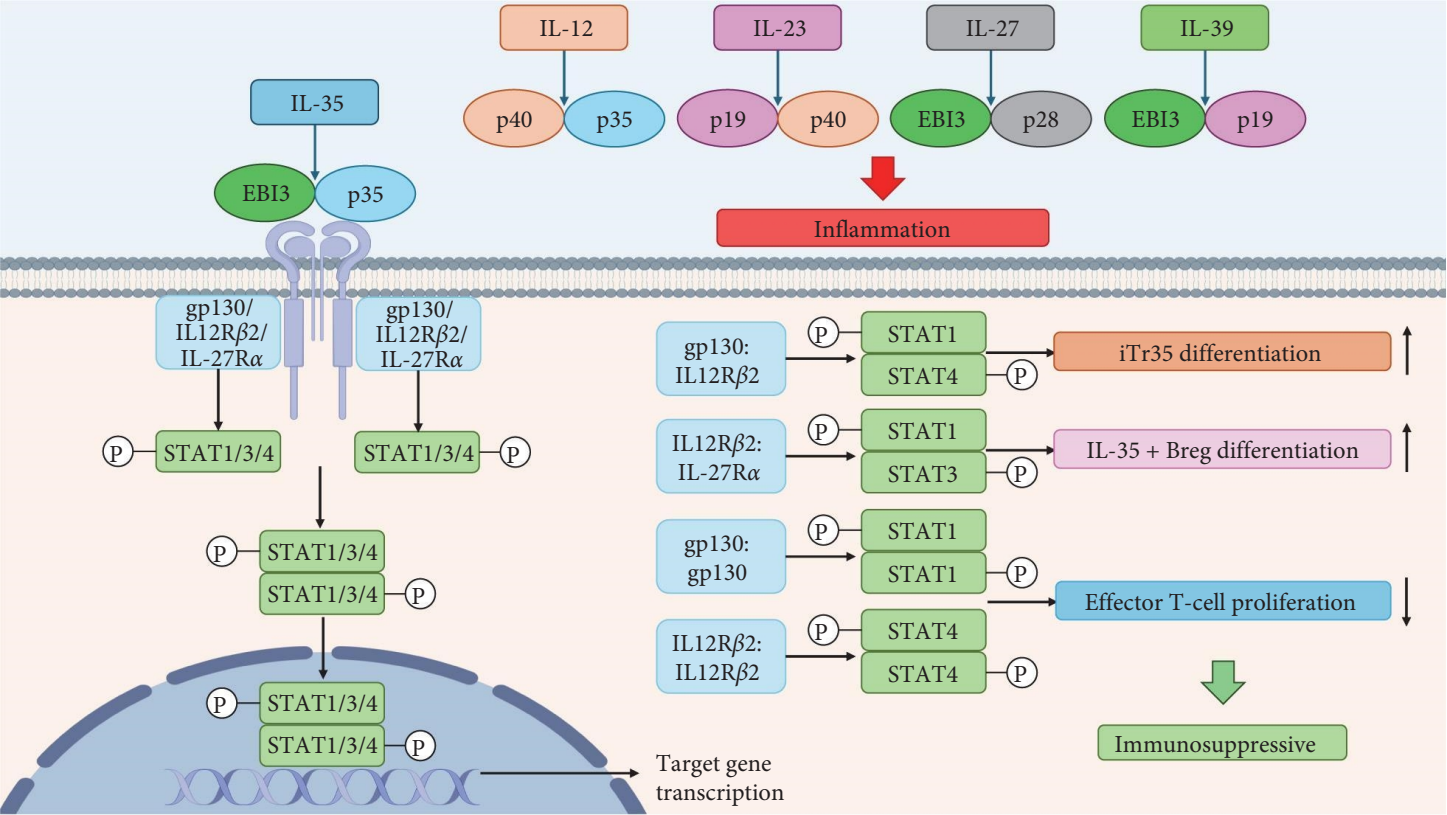
IL-35, a member of the IL-12 family, consists of EBI3 and p35 subunits. While other IL-12 family members (IL-12, IL-23, IL-27, IL-39) are proinflammatory, IL-35 exerts immunosuppressive effects. It activates receptors (gp130/IL-12R*β*2/IL-27R*α*) on the cell membrane, recruiting STAT proteins (STAT1, STAT3, STAT4) to form dimers that enter the nucleus to regulate gene transcription. EBI3, Epstein–Barr virus induced 3; IL, interleukin.

Upon stimulation by members of the IL-12 family, the cell membrane undergoes structural alterations in the surface receptor, leading to the activation of Janus kinases (JAKs) such as Jak1, Jak2, and Tyk2. These JAKs then phosphorylate the receptor, creating phosphotyrosine docking sites that attract specific signal transduction factor STAT molecules. Following recruitment to these sites, STAT is phosphorylated, resulting in the formation of specific dimers that translocate to the nucleus. Once in the nucleus, these dimers bind to the promoter regions of target genes and modulate their transcription [50, 53–55]. For instance, IL-12 facilitates Th1 cell differentiation primarily by stimulating the formation of p-STAT4:p-STAT4 dimers [56]; IL-23 promotes Th17 cell differentiation mainly through activation of p-STAT3:p-STAT3 dimers [57, 58]; IL-27 triggers the activation of p-STAT1 and p-STAT3 to create homo- or heterodimers, which produce immunomodulatory effects and demonstrate both proinflammatory and suppressive immune responses in various diseases [59–62]; IL-39 activates p-STAT1:p-STAT3 heterodimers and induces proinflammatory effects. Due to the specificity of the IL-35 receptor [48], IL-35 can initiate the formation of p-STAT1:p-STAT3, p-STAT1:p-STAT4, p-STAT1:p-STAT1, and p-STAT4:p-STAT4, although other combinations of STAT proteins may also contribute to its downstream biological functions [8, 9, 15] (Figure 2).

### 3.3. The Function of IL-35

IL-35, a crucial immunosuppressive factor within the IL-12 family [63], has been extensively researched for its role in mitigating inflammatory responses in various diseases, including ulcerative colitis, systemic lupus erythematosus, acute kidney injury, influenza A and hepatitis B [64–68]. Its immunosuppressive function is primarily mediated by Tregs, Bregs, and macrophages through the secretion of anti-inflammatory cytokines.

Initially, IL-35 demonstrates immunosuppressive properties through the differentiation of T cells and B cells [14, 15, 69]. Collison et al. [14] observed elevated expression of the IL-35 subunits EBI3 and p35 in Tregs, while its expression was absent in quiescent or activated effector T cells (Teff). Furthermore, IL-35 secreted by Tregs can promote autocrine IL-35 production, thereby amplifying the immunosuppressive capabilities of Tregs [14]. Collison et al. [69] demonstrated that IL-35 not only promotes IL-35 production in mouse CD4^+^Foxp3^+^Tregs, but also facilitates the development of IL-35-induced Tregs (iTR35 cells) in CD4^+^Foxp3^−^T cells [69]. Additionally, Shen et al. [15] identified that IL-35 enhances the phosphorylation of STAT1 and STAT3 via the IL-12R*β*2:IL-27R*α* receptor, resulting in increased IL-35 production in mouse B cells and the differentiation of B cells into IL-35-secreting B cells. Ma et al. [70] found that IL-35 induces the production of iTR35 in human CD4^+^ T cells by promoting p-STAT1:p-STAT3 synthesis.

Furthermore, IL-35 demonstrates a multifaceted role in immune regulation by promoting Treg differentiation and proliferation while concurrently inhibiting the differentiation and proliferation of T helper cell subsets Th1, Th2, and Th17. IL-35 also suppresses the secretion of key cytokines associated with these subsets, including IFN-*γ*, IL-4, IL-5, IL-13, and IL-17 [71–74]. Moreover, IL-35 exerts inhibitory effects on both CD4^+^ and CD8^+^ T cell proliferation [75, 76], as well as macrophage polarization toward the proinflammatory M1 phenotype, favoring polarization toward the anti-inflammatory M2 phenotype and ultimately dampening inflammatory responses [9]. An increasing body of research has demonstrated that various cell types, including dendritic cells, smooth muscle cells, neutrophils, and tumor cells, are capable of secreting IL-35 during inflammatory conditions, leading to diverse physiological effects [8, 9, 77, 78]. Furthermore, IL-35 exhibits anti-inflammatory properties by stimulating the release of anti-inflammatory factors such as IL-10, TGF-*β*, and IL-23 from target cells [9, 16, 79] (Figure 2).

However, the potential inhibitory effects of IL-35 on other proinflammatory cytokines within the IL-12 family remain uncertain. Shared subunits EBI3 with IL-27 and IL-39, and p35 with IL-12, suggest that IL-35 could competitively inhibit the production of IL-12, IL-27, and IL-39. Nevertheless, research indicates that the formation of IL-35 dimers is not mediated by IL-12A in IL-12 and EBI3 in IL-27, suggesting that structural competitive inhibition may not be a mechanism by which IL-35 inhibits IL-12 and IL-27 production [80]. Li et al. [81] proposed that the interaction between IL-35 and its receptor leads to the inhibition of proinflammatory cytokine signaling, including IL-6, IL-12, and IL-27 [81]. This inhibition may be attributed to the competitive binding of IL-35 and other members of the IL-12 family to the same receptor chain, resulting in an immunosuppressive physiological response [82]. However, it has also been observed that IL-35 can dose-dependently stimulate the production of inflammatory factors and chemokines, such as IL-6, IL-1*β*, and MCP-1, by peripheral blood mononuclear cells (PBMCs) [83]. This phenomenon may be attributed to the interaction of the IL-35 subunit, EBI3, with gp130-expressing cells, resulting in both proinflammatory and anti-inflammatory effects. The binding of the EBI3 subunit to IL-6 and subsequent transmembrane signaling is believed to enhance the production of chemokines that promote proinflammatory responses. Consequently, Chehboun et al. [84] recommend vigilance in maintaining the structural integrity of IL-35 when considering its use as a therapeutic agent to prevent potential proinflammatory outcomes.

### 3.4. IL-35 and Endothelial Dysfunction

**Figure 3:**
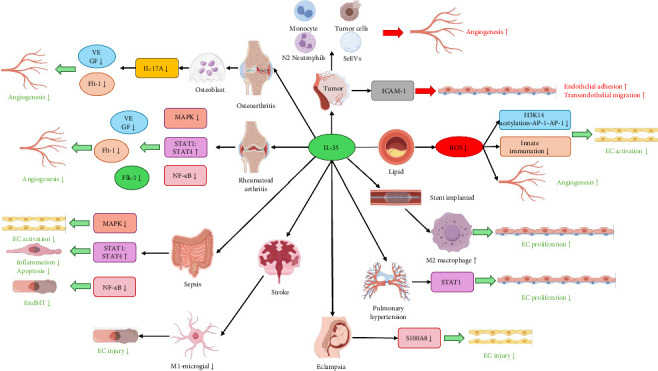
The role of IL-35 in endothelial cells and endothelial function across various diseases. IL-35 inhibits endothelial cell activation and damage induced by various stimuli, such as high-fat diets, lipopolysaccharides, and inflammatory factors, thereby alleviating endothelial dysfunction. Additionally, IL-35 modulates the expression of VEGF and its receptors in various cells within the disease microenvironment, regulating angiogenesis. IL-35, interleukin-35; VEGF, vascular endothelial growth factor.

Recent studies have demonstrated that both exogenous and endogenous IL-35 can modulate various cellular functions in ECs, including proliferation, apoptosis, inflammation, adhesion, migration, mesenchymal transition, and neovascularization, either directly or indirectly (Figure 3).  Table 1 summarizes studies on the effects of IL-35 on endothelial function in vascular diseases, such as atherosclerosis, delayed post-stent endothelialization, pulmonary arterial hypertension (PAH), pre-eclampsia (PE), ischemic stroke, and peripheral arterial disease (PAD).  Table 2 presents IL-35's impact on endothelial function in tumors, while Table 3 focuses on its effects in RA. Other conditions, including sepsis, OA, and transfusion-related lung injury, are not included in the tables due to limited study numbers but are discussed in detail in the text. The following sections will provide a comprehensive examination of how these processes contribute to various pathological states.

#### 3.4.1. Atherosclerosis

Atherosclerosis is currently acknowledged as an autoimmune condition [89, 90] believed to result from prolonged injury to the vascular endothelium caused by multiple risk factors [91, 92]. Endothelial dysfunction serves as an initial indication of atherosclerosis, with damage to the endothelium activating various factors that contribute to the advancement of the disease, ultimately leading to further endothelial dysfunction [93, 94]. Shao et al. [95] have shown that IL-35 hinders atherosclerosis by enhancing the production of CD4^+^Foxp3^+^Tregs through the facilitation of CCR5 expression [95]. Furthermore, Bhansali et al. [96] demonstrated that exogenous IL-35 altered miRNA expression in macrophages derived from individuals with coronary artery disease, leading to a reduction in oxidized low-density lipoproteins (ox-LDLs)-induced atherosclerosis. Additionally, IL-35 was found to mitigate high-fat-induced endothelial dysfunction in addition to its atherosclerosis-inhibiting effects. The activation of ECs by lysophosphatidylcholine (LPC) serves as a crucial initial event in the pathogenesis of atherosclerosis. During the early stages of hyperlipidemia, LPC triggers EC activation, leading to the production of ICAM-1. This molecule facilitates the adhesion and migration of leukocytes to the endothelium, thereby promoting the development of atherosclerosis [97–99]. Additionally, LPC induces the innate immune differentiation and training of immune characteristics in ECs [100].

Li et al. [99] illustrated that LPC induces an increase in IL-35 expression in the serum, leading to heightened IL-35 receptor expression in aortic cells, thereby preventing LPC-induced monocyte adhesion and ICAM-1 expression. Subsequent investigations revealed that IL-35 inhibits histone H3 lysine 14 acetylation stimulated by mitochondrial reactive oxygen species (mtROS), with this acetylation promoting EC ICAM-1 expression through activator protein-1 (AP-1) [100]. Consequently, IL-35 emerges as a pivotal factor in suppressing EC activation induced by elevated lipid levels [18]. Another investigation suggested that IL-35 inhibits LPC-induced intercellular adhesion, cellular activation, cytokine, and receptor activation through the inhibition of mtROS production and reverse innate immune signaling pathways such as Toll-like receptors and nod-like receptors. However, IL-35 does not affect trained immune signaling pathways such as glycolysis and cholesterol homeostasis pathways [17]. These results indicate that IL-35 may effectively suppress high-fat-induced endothelial dysfunction and the progression of atherosclerosis.

#### 3.4.2. Delayed Post-Stent Endothelialization

Delayed post-stent endothelialization is a pathological condition characterized by inadequate coverage of the stent surface by newborn ECs following stent implantation, thereby increasing the risk of in-stent restenosis and thrombosis [101]. This phenomenon is primarily attributed to EC dysfunction and limited proliferative and regenerative capabilities [102, 103]. Macrophage polarization toward the M1 phenotype is implicated in the production of peroxides, leading to endothelial dysfunction and hindering stent coverage and endothelial repair [104].

Liu et al. found a significant elevation in serum IL-35 levels and decreased IL-1*β* levels in patients with covered stents compared to those with uncovered stents. Their investigation involved the examination of human PBMC treated with IL-35 and/or IFN-*γ* + LPS for 24 h, revealing that IL-35 facilitates the polarization of macrophages toward the M2 phenotype via the IL-12R*β*2:gp130/p-STAT1:p-STAT4 signaling pathway. Subsequently, they demonstrated that within a model of TNF-*α*-induced endothelial dysfunction, IL-35 exhibited beneficial effects on EC proliferation and angiogenesis, suppression of apoptosis, enhancement of secretion of IL-10 and TGF-*β*, inhibition of IL-12 secretion, and attenuation of NF-*κ*B and I*κ*B*α* phosphorylation through the induction of macrophage polarization toward the M2 phenotype, ultimately resulting in enhanced endothelial function [9]. Consequently, IL-35 may have the potential to ameliorate inadequate endothelialization following stent placement by promoting macrophage polarization toward the M2 phenotype.

#### 3.4.3. PAH

PAH is a severe condition characterized by elevated pressure in the pulmonary arteries, resulting in potential right ventricular overload and progression to right heart failure [105]. ECs play a crucial role in the pathogenesis of PAH, with abnormalities in endothelial growth factors (e.g., vascular endothelial growth factor [VEGF]) and endothelial generating factors (e.g., NO) observed in PAH patients [90]. These variables have the potential to impact the functionality of ECs with regard to angiogenesis, migration, and vasoconstriction, ultimately resulting in endothelial dysfunction [106, 107]. Consequently, the suppression of angiogenesis in ECs is considered a promising therapeutic approach.

Wan et al. [6] observed elevated levels of EBI3, IL-12A, and serum IL-35 in the lung tissues of mice afflicted with PAH compared to their healthy counterparts. The transplantation of Tregs effectively ameliorated pulmonary hypertension, with a reduced therapeutic efficacy observed in mice subjected to EBI3 knockout or treated with IL-35 antibodies. Furthermore, both in vitro and in vivo experiments demonstrated that IL-35 suppressed EC proliferation under hypoxic conditions, and this effect was attenuated by blocking STAT1. Subsequent investigations revealed that Tregs-derived IL-35 suppressed the excessive proliferation of pulmonary artery ECs via modulation of the gp130/STAT1 signaling pathway [6].

#### 3.4.4. PE

PE is a hypertensive disorder that poses a significant risk to the health of pregnant women, characterized by systemic maternal endothelial dysfunction [108–110]. Elevated levels of various factors, including TNF-*α*, IL-6, lipid peroxides, cellular debris, and ox-LDL, are commonly observed in the bloodstream of individuals with PE [111, 112]. These factors contribute to the generation of ROS and peroxides, ultimately leading to EC damage [113, 114].

Li et al. discovered a significant decrease in the percentage of iTR35 cells in the peripheral blood of patients with PE, along with reduced serum IL-35 concentrations. They also observed that IL-35 modulated the calcium-binding protein S100A8, which plays a role in scavenging reactive oxygen species (ROS) and reducing oxidative stress [115]. This modulation inhibited the generation of ROS induced by PE serum, mitigated the decrease in matrix metalloproteinases (MMPs), and prevented apoptosis in human umbilical vein ECs (HUVECs) [20]. Therefore, IL-35 may serve as a novel biomarker with both theoretical and practical implications for the prenatal screening and treatment of PE patients.

#### 3.4.5. Ischemic Stroke

Ischemic stroke results from a reduction in cerebral blood flow due to the obstruction of cerebral blood vessels [116]. Following treatment with recombinant tissue-type plasminogen activator or interventional thrombolysis, patients commonly experience postischemic reperfusion injury, leading to harm to cerebral microvascular ECs and compromising the integrity of the blood–brain barrier (BBB) [117]. This ultimately diminishes the effectiveness of flow-restoring therapy [118]. Furthermore, under ischemic and hypoxic conditions, there is a prompt activation of microglia leading to the polarization into M1 or M2. Unregulated activation of M1 microglia can cause harm to healthy neurons, diminish the viability of impaired neurons, and affect ECs negatively. Conversely, M2 microglia have the capability to suppress the inflammatory reaction and aid in the restoration of brain injury [119, 120].

Liu et al. [21] constructed a murine model of middle cerebral artery occlusion and administered intraventricular injections of recombinant IL-35. They demonstrated that IL-35 facilitates the transformation of microglia into M2 by suppressing ROS generation, NF-*κ*B phosphorylation, and HIF1-*α* production, ultimately decreasing their viability in the presence of oxygen and reducing their detrimental impact on ECs during oxygen-glucose deprivation-reperfusion (OGD-R) conditions [21]. The research group further observed that IL-35 suppressed ROS generation, p38 phosphorylation, and the expression of inflammatory cytokines IL-1*β* and IL-18 while enhancing the expression of tight junction proteins ZO-1 and occludin in mouse cerebral microvascular ECs subjected to OGD-R. Additionally, IL-35 was found to inhibit caspase-1 production via the ROS/TXNIP signaling pathway [22]. These findings suggest that IL-35 exerts a protective effect on ECs against OGD-R-induced damage, either through direct or indirect mechanisms.

#### 3.4.6. PAD

PAD is characterized by reduced blood flow to peripheral limbs and organs as a result of stenosis caused by atherosclerosis in the arteries of the lower extremities [121, 122]. A prevalent symptom is pain during movement of the lower extremities, and PAD is closely linked to coronary heart disease and stroke. Despite recommendations advocating surgical intervention for advanced PAD, a significant number of patients with advanced PAD are unable to achieve favorable outcomes due to surgical challenges [123, 124].

Recent research indicates that IL-35 plays a significant role in PAD treatment. Fu et al. discovered that in a mouse model of hindlimb ischemic disease, there was an increase in the expression of IL-35 and its receptor subunit, IL-12R*β*2, in muscle, while the expression of gp130 remained unchanged. Furthermore, they observed that IL-35 hindered the early injury/inflammatory vascular regeneration induced by hind limb ischemia (HLI) by reducing the expression of ROS and enhancing the expression of anti-angiogenic extracellular matrix remodeling proteins yet maintained the vascular regenerative function in the later stages of HLI [5]. These findings offer a novel perspective and insight into the potential therapeutic role of IL-35 in the treatment of PAD.

#### 3.4.7. Tumors

ECs are crucial participants in the process of tumor development, as they are stimulated by angiogenic proteins secreted by the cells in the tumor microenvironment, leading to the formation of new blood vessels [125, 126]. Additionally, these cells release cytokines that enhance EC activation and permeability, facilitating the migration of tumor cells across vessel walls [127, 128]. IL-35 has been implicated in tumorigenesis and progression in numerous research studies. Recent research has demonstrated that Tregs are capable of producing IL-35, a cytokine that hinders the antitumor functions of lymphocytes, enhances the accumulation of myeloid cells, stimulates angiogenesis, and expedites tumor advancement [129, 130]. Furthermore, IL-35 can expedite tumor progression by modulating the physiological functions of tumor cells and various other cell types within the tumor microenvironment, including ECs and tumor-associated macrophages, thereby influencing processes such as cell proliferation, apoptosis, and angiogenesis, ultimately impacting tumor development [85, 87, 131]. Current research indicates that IL-35 within the tumor microenvironment typically does not directly impact ECs. Instead, IL-35 influences the biological activities of ECs, including activation, migration, adhesion, and neovascularization, through interactions with other cells such as tumor cells [87], monocytes [19], and neutrophils [78] present in the tumor microenvironment.

Wang et al. [132] demonstrated a notable augmentation in vascular neovascularization within tumor tissues following the subcutaneous injection of mouse plasmacytoma J558 cells and mouse melanoma B16 cells overexpressing IL-35 in mice [132]. Zhu et al. [86] observed that IL-35 facilitated the proliferation of mouse prostate tumors and the formation of microvessels, while the administration of IL-35 antibodies resulted in contrasting effects [86]. Li et al. [87] discovered that IL-35 was upregulated in human gastric cancer tissues and exhibited a positive correlation with microvessel density. While rhIL-35 did not directly impact the angiogenesis of HUVEC in vitro tube-forming assays, the supernatant from gastric cancer cells overexpressing IL-35, EBI3, and IL-12A notably enhanced HUVEC angiogenesis. Subsequent investigations revealed that IL-12A overexpression stimulated the expression of angiogenesis-associated proteins TIMP1, IGFBP1, and PAI1 in tumor cells. IL-12A, a component of IL-35, may be responsible for stimulating tumor cells to produce angiogenic proteins [87].

In addition to tumor cell-derived IL-35 promoting angiogenesis, Huang et al. indicated that IL-35 originating from pancreatic ductal adenocarcinoma (PDAC) cells stimulated the GP130/STAT1 signaling pathway, resulting in the formation of p-STAT1:p-STAT4. This activation subsequently enhances the expression of intercellular adhesion molecule 1 (ICAM1) in PDAC cells, which facilitates the adhesion and transendothelial migration of ECs in tumor tissues by forming an ICAM1-fibrinogen-ICAM1 bridge [8]. Subsequent research demonstrated a notable increase in the quantity of microvessels within PDAC tissues exhibiting high levels of IL-35 expression. Moreover, the upregulation of IL-35 in tumor cells facilitated the recruitment of monocytes to PDAC tissues through the chemokine CCL5. IL-35 also induced the upregulation of angiogenesis-related genes (CXCL1 and CXCL8) in monocytes, consequently promoting angiogenesis in PDAC tissues. These effects of IL-35 are mediated through the activation of STAT1 and STAT4 phosphorylation via the IL12RB2:gp130 receptor, leading to the transcription of CCL5 in PDAC and CXCL1 and CXCL8 in monocytes [19]. Additionally, Zou et al. revealed that the overexpression of IL-35 in neutrophils, rather than exogenous IL-35, stimulated the production of G-CSF and IL-6 by neutrophils, induced polarization toward the N2 subtype associated with tumor growth, upregulated MMP-9 and Bv8 expression, downregulated TRAIL expression, and bolstered the proendothelial angiogenic capabilities of neutrophils [78]. In a separate study, Liu et al. illustrated that IL-35 stimulated the generation of exosomes carried IL-35 in breast cancer cells, termed IL-35-sEVs, which subsequently enhanced HUVEC cell proliferation and angiogenesis through the activation of the CALM1/Ras/Raf/MEK/ERK signaling pathway [88].

Previous research has indicated that exogenous IL-35 typically does not exert direct effects on ECs to stimulate neovascularization but instead elicits its impact through indirect mechanisms within the tumor microenvironment. Specifically, IL-35 enhances the proliferation, adhesion, and neovascularization capabilities of ECs by inducing the secretion of angiogenic proteins and adhesion molecules by tumor cells, monocytes, and neutrophils. While the precise role of IL-35 in tumorigenesis and progression remains incompletely understood, its utility as a promising biomarker for cancer prognosis and therapy warrants further investigation. Evidence suggests that continuous injection of anti-IL-35 can effectively impede tumor neovascularization, underscoring its potential clinical significance [132].

Nevertheless, certain research studies have indicated that IL-35 may possess the capability to induce apoptosis in tumor cells. Long et al. conducted in vitro experiments, which demonstrated that the upregulation of IL-35 impeded the proliferation of diverse human tumor cells, triggered cell cycle arrest in the G1 phase, and facilitated apoptosis through the upregulation of Fas expression and downregulation of cyclinD1, survivin, and Bcl-2 expression [131]. Additionally, a study illustrated that upregulation of IL-35 in HepG2 cells derived from individuals with advanced hepatocellular carcinoma resulted in a notable increase in the expression levels of HLA-ABC and CD95, suppression of MMP-2 and MMP-9 activity, as well as a reduction in cell migration, invasion, and colony formation capabilities [133]. The observed discrepancies in these outcomes could potentially be attributed to the mode of action of IL-35, varying stages of tumor progression, and distinct cellular microenvironments.

#### 3.4.8. Sepsis

Sepsis is a critical medical condition characterized by a dysregulated immune response to infection, resulting in organ dysfunction [134]. In sepsis, ECs become activated, leading to an increase in the expression of various adhesion molecules such as ICAM-1, VCAM-1, E-selectin, P-selectin, and vWF [135]. While moderate activation of ECs plays a role in controlling bacterial spread and facilitating the recruitment and elimination of leukocytes, severe or prolonged alterations in endothelial phenotype can result in compromised microcirculatory blood flow, insufficient tissue perfusion, and ultimately, organ failure [136, 137]. It is crucial to consider therapeutic strategies that modulate endothelial innate immune responses in sepsis patients.

A series of studies have shown that IL-35 can improve endothelial dysfunction caused by sepsis. Sha et al. discovered that IL-35 levels are heightened in the serum of sepsis patients. Using a sepsis mouse model, they demonstrated that IL-35 suppresses VCAM-1 expression via the IL-12R*β*2:gp130 heterodimeric receptor and inhibits the MAPK-AP-1 signaling pathway [33]. Our research further indicated that IL-35 upregulates its own expression in mouse aortic tissues while reducing the expression of adhesion molecules VCAM-1 and ICAM-1, as well as inflammatory factors IL-6 and CXCL15. IL-35 demonstrated the ability to suppress LPS-induced apoptosis in HUVEC, stimulate their proliferation, and inhibit EC activation through the inducing p-STAT1:p-STAT4 synthesis [16].

Moreover, lipopolysaccharide not only triggers EC activation but also initiates endothelial-to-mesenchymal transition (EndMT) [138, 139], a process in which ECs adopt mesenchymal cell characteristics, resulting in the loss of their original EC functions. This transition is marked by a reduction in the expression of EC markers CD31 and E-cadherin and an elevation in the expression of mesenchymal cell markers *α*-SMA, vimentin, and fibronectin [140, 141]. Our study revealed that IL-35 effectively mitigated the reduced expression of eNOS and CD31 in the aorta of septic mice, as well as counteracted the LPS-induced decline in the proliferative ability of HUVEC. Additionally, IL-35 inhibited ROS production induced by LPS and suppressed the EndMT by hindering NF-*κ*B phosphorylation and its translocation into the nucleus [32]. These findings indicate that IL-35 shows promise as a therapeutic target for alleviating sepsis-induced endothelial injury.

#### 3.4.9. RA

RA is a persistent condition that results in diminished physical function and quality of life, as well as imposes social burdens, including functional disability and decreased work capacity. Consequently, the exploration of novel therapeutic approaches is imperative [142]. Angiogenesis plays a pivotal role in the early stages of RA pathogenesis, facilitating the proliferation of synovial tissue and the development of vascular abnormalities. In RA, the activation of the angiogenic switch and subsequent angiogenesis can be further stimulated by inflammation, immune imbalance, and hypoxia. The resultant vascular system recruits leukocytes, perpetuates immune imbalance, and exacerbates inflammation [143–145]. Numerous research studies have demonstrated an increase in serum IL-35 expression among individuals with RA, as well as an association between IL-35 gene polymorphisms and RA susceptibility [146, 147]. IL-35 plays a role in modulating the pathogenesis of RA and is correlated with disease severity [147]. Its therapeutic effects in alleviating RA symptoms are attributed to its inhibition of fibroblast-like synoviocyte (FLS) proliferation, angiogenesis, and bone destruction, primarily through the promotion of Tregs proliferation and the suppression of Th17 cells differentiation [146].

IL-35 has been identified as a regulator of angiogenesis in RA. Lu et al. conducted a series of investigations in this area. First, they collected RA synovial tissue explants from patients undergoing RA surgery. Coculturing these explants incubated with IL-35 and HUVECs effectively inhibits VEGF-induced HUVEC wound healing, chemotactic migration, cell adhesion, and tube formation through the Ang2/Tie2 pathway [23]. Additionally, a collagen-induced arthritis (CIA) model was established in mice, who were subsequently administered daily injections of 2 µg of rmIL-35 for 10 days starting from day 24 of successful model construction. Tests conducted 40 days posttreatment demonstrated that IL-35 suppressed the expression of VEGF and its receptors Flt-1 and Flk-1, as well as the inflammatory factor TNF-*α* in synovial tissues [24]. Following this, Lu et al. isolated FLS from mice with CIA and exposed them to varying concentrations of IL-35. The researchers observed a dose-dependent inhibition of VEGF, FGF-2, endostatin, TNF-*α*, and IL-6 expression in FLS, along with an increase in endostatin expression. These effects were mediated through the STAT1 signaling pathway [25]. Houshmandi et al. [26] found that in the RA model rats, rhIL-35 demonstrated no significant impact on inflammatory lesions and neovascularization at arthropathic lesions but exhibited a significant inhibitory effect on inflammatory lesions and neovascularization at lesions with p35 inhibitors [26]. These findings suggest that endogenous IL-35 plays a crucial role in stimulating inflammatory proliferation and angiogenesis at RA lesions. Interestingly, Lu et al. [27] made an intriguing discovery that treatment of mouse embryonic osteoblast precursor cells (MC3T3E1 cells) with 20 ng/ml of TNF-*α*, simulating the effects of RA on osteoblasts, followed by treatment with varying concentrations of IL-35, resulted in a concentration-dependent promotion of MC3T3E1 cell proliferation, upregulation of VEGF and its receptor Flt-1 and Flk-1 expression, and inhibition of apoptosis [27].

The results of the aforementioned studies indicate variations in the influence of IL-35 on cellular secretion of angiogenic proteins at RA lesions, potentially attributable to variances in cell types and experimental models utilized in vitro or in vivo. Hence, additional research is required to elucidate the potential therapeutic role of IL-35 in RA.

#### 3.4.10. Osteoporosis and OA

Osteoporosis, the most prevalent bone metabolism disorder, is pathologically defined by a reduction in bone microstructure and density. The condition arises from an imbalance in the activities of osteoclasts and osteoblasts, leading to heightened osteoclastogenesis and elevated bone resorption [148]. Furthermore, neovascularization around osteoclasts is a crucial factor in the development of osteoporosis [149, 150]. A study demonstrated that IL-35 suppresses TNF-*α*-induced osteoclastogenesis and facilitates apoptosis [151]. VEGF and its receptor Flt1-mediated angiogenesis provide essential nutrients to osteoblasts and osteoclasts, although excessive VEGF expression can result in heightened osteoclast activity [152]. Zhang et al. [153] observed that IL-35 inhibits the expression and function of VEGF and Flt-1 in osteoclasts via the Th17/IL-17 pathway, ultimately promoting apoptosis in these cells [153].

OA, or degenerative joint disease, is marked by the deterioration of articular cartilage. In a healthy state, articular cartilage is a nonvascular tissue reliant on nutrients from synovial fluid [154]. In OA, cartilage breakdown can prompt neovascularization near the lesion, resulting in the growth of new blood vessels from the subchondral layer into the cartilage interior. This process can contribute to arthritis development, attract inflammatory cells, exacerbate the inflammatory response, increase vascular permeability, and thereby leading to edema [155, 156]. Yang et al. [157] established an in vitro model of OA by inducing SW1353 (human chondrosarcoma cells) with IL-1*β* and observed that IL-35 suppressed the release of the proangiogenic factor Ang2 in SW1353 cells through the P38 MAPK signaling pathway [157]. Additionally, they discovered that IL-35 also downregulated the expression of VEGFA and its receptor FLT1, PDGF-BB and its receptor PDGFR-*α*, and PDGFR-*β* in IL-1*β*-stimulated SW1353 cells via the Th17/IL-17 pathway [158].

In conditions such as osteoporosis and OA, IL-35 suppresses angiogenesis in ECs within the joint microenvironment predominantly through the inhibition of osteoclasts and chondrocytes' secretion of angiogenic factors.

#### 3.4.11. Transfusion-Associated Acute Lung Injury (TRALI)

TRALI is a significant complication of transfusion characterized by acute respiratory distress, hypoxemia, and noncardiogenic pulmonary edema, typically presenting within 6 h posttransfusion [159, 160]. Presently, there is a lack of efficacious clinical interventions for TRALI. The pathophysiology of TRALI is mediated by antileukocyte antibodies or biological response modifiers (BRMs) that induce TRALI via activation of the EC pathway [161].

Qiao et al. discovered that the administration of 100 µg/kg of recombinant IL-35 via intravenous injection for three consecutive days resulted in a notable reduction in the expression of E-selectin, P-selectin, and ICAM-1 in both lung tissue and serum, as well as a decrease in serum levels of IL-6, IFN-*γ*, and TNF-*α* in a mouse model of TRALI. This intervention effectively mitigated pulmonary vascular EC activation and inflammatory response, leading to a significant alleviation of TRALI-induced pulmonary edema [162].

## 4. Conclusion

IL-35, a member of the IL-12 cytokine family, demonstrates primarily immunosuppressive effects and is upregulated in response to various acute and chronic stimuli. It is mainly secreted by Tregs and Bregs. IL-35 plays a regulatory role in EC functions such as proliferation, apoptosis, inflammatory response, adhesion, migration, mesenchymal transition, and angiogenesis through diverse mechanisms. The divergent impact of IL-35 on EC adhesion and neovascularization in various diseases may be attributed to factors such as the method of IL-35 administration, dosage, treatment duration, and the progression of the specific disease being investigated. Despite this variability, aberrant expression of IL-35 has been observed in a range of conditions, including coronary artery disease, tumors, and autoimmune disorders, suggesting its potential as a therapeutic target for these ailments. Subsequent research endeavors should focus on elucidating the precise mechanisms underlying IL-35's efficacy in diverse disease states and investigating its potential for clinical utility.

## Figures and Tables

**Table 1:** Studies reporting the effects of IL-35 on endothelial function in vascular diseases, including atherosclerosis, delayed post-stent endothelialization, PAH, PE, ischemic stroke, and PAD.

Author/year ref.	Type of study	Cohort	Aims	Findings
Li et al. 2018 ATVB [18].	Cell model (HAEC) Mice model	Control LPC LPC + IL-35 ApoE-/-HF ApoE-/-HF + IL−35	Investigate whether IL-35 inhibits atherogenic lipid-induced EC activation and atherosclerosis	IL-35 is induced during atherosclerosis development and inhibits mtROS- H3K14 acetylation-AP-1-mediated EC activation.

Li et al. 2020 Redox Biology [17].	Cell model (HAEC)	Control LPC LPC + IL−35	Investigate how IL−35 blocks atherogenic lipid and LPS-induced EC activation and whether mtROS differentiate EC activation from trained immunity	IL-35 may reverse mtROS-mediated innate immune signaling pathways in HAECs while sparing metabolic reprograming and trained immunity, which may not fully depend on mtROS.

Liu et al. 2019 Clinical Science [9].	Cell model (macrophages) (HUVEC) Rabbits model	Control IL-35+si-STAT1/4 IL-35+anti-IL-12R*β*2 IL-35+anti-GP130 Control TNF-*α* TNF-*α*+cocultured with IL-35-treated macrophages SESs implanted SESs implanted + IL-35	Investigate the relationship between IL-35 and early strut coverage	IL-35 activated an anti-inflammatory M2-like macrophage phenotype via STAT1/4, enhancing endothelial proliferation and improving dysfunction with early strut coverage in SES-treated rabbits.

Wan et al. 2021 Ann Transl Med [6].	Cell model (HUVEC) Mice model	Control Hypoxia+IL-35 Hypoxia+IL-35+anti-GP130 Hypoxia+IL-35+anti-IL12R*β*2 Hypoxia+IL-35+STAT1 inhibitor PH PH + Anti-IL-35 Ab	Investigate the mechanism of IL-35 and its downstream molecules in the development of pulmonary hypertension	Tregs-derived IL-35 alleviates pulmonary hypertension by inhibiting EC proliferation via STAT1, reversing pulmonary vascular remodeling.

Li et al. 2020 Hypertens Pregnancy [20]	Cell model (HUVEC)	PE serum PE serum+IL-35 PE serum+IL-35+S100A8 shRNA	Investigate the protective effects of IL-35 on HUVECs injured by PE patient serum	IL-35 inhibited PE serum-induced HUVEC apoptosis and ROS levels by S100A8.

Qian et al. 2022 Ann Transl Med [21].	Cell model (bEnd.3) Mice model	Control OGD-R OGD-R+IL-35 Sham MCAO MCAO + IL-35	Investigate the effects of IL-35 on BBB dysfunction in ischemic stroke	IL-35 exerts a protective effect on the BBB by targeting the ROS/TXNIP/caspase-1 pathway in cerebral ischemia-reperfusion (I/R) injury.

Liu et al. 2022 Transl Pediatr [22].	Cell model (HUVEC) Rats model	Control Cocultured with microglia culture medium +PBS Cocultured with IL-35-treated microglia culture medium +PBS Sham HI HI + IL-35	Investigate the effect of IL-35 treatment on neonatal rats with hypoxic-ischemic brain injury	IL-35 reversed M1-microglial polarization-induced endothelial injury and suppressed HIF-1 *α* and NF-*κ*B signaling in vivo and in vitro.

Fu et al. 2020 Front lmmunol [5].	Cell model (HMVEC) Mice model	Control IL-35 FGF2 IL-35+FGF2 HLI HLI + IL-35	Investigate how IL-35 regulates ischemia-induced angiogenesis in peripheral artery diseases	IL-35 reduced ROS expression and promoted antiangiogenic matrix remodeling in early HLI while preserving vascular regeneration in later stages.

*Note:* The references are listed in the order of progression in the text, as shown in the PRISMA flowchart.

Abbreviations: BBB, blood–brain barrier; EC, endothelial cell; HLI, hind limb ischemia; HUVECs, human umbilical vein endothelial cells; I/R, ischemia-reperfusion; IL-35, interleukin-35; LPC, lysophosphatidylcholine; mtROS, mitochondrial reactive oxygen species; OGD-R, oxygen-glucose deprivation-reperfusion; PAD, peripheral arterial disease; PAH, pulmonary arterial hypertension; PE, pre-eclampsia; ROS, reactive oxygen species; Tregs, regulatory T cells.

**Table 2:** Studies reporting the effects of IL-35 on endothelial function in tumors.

Author/year ref.	Type of study	Cohort	Aims	Findings
Wang et al. 2013 J Immunol [85].	Cell model (J558, B16) Mice model	Control IL-35 J558-Control J558-IL-35 B16-Control B16-IL-35	Investigate the roles of tumor-derived IL-35 in tumorigenesis and tumor immunity	Tumor-derived IL-35 increases CD11b+Gr1+ myeloid cell accumulation in the tumor microenvironment and, thereby, promotes tumor angiogenesis.

Zhu et al. 2020 Cancer Cell Int [86].	Mice model	Control IL-35 IL-35 NA Scramble	Investigate the effects of IL-35 and its immunoregulatory effect on PCA	IL-35 facilitates the proliferation of mouse prostate tumors and the formation of microvessels.

Li et al. 2020 FEBS Open Bio [87].	Cell model (HUVEC)	Control IL-35 s-IL-35 pc-IL-12A pc-EBI3 sh-IL-12A sh-EBI3	Investigate the role of IL-35 in the angiogenesis of gastric cancer	IL-35 did not directly affect HUVEC angiogenesis in tube-forming assays, but the supernatant from gastric cancer cells overexpressing IL-35, EBI3, and IL-12A significantly enhanced it.

Huang et al. 2017 Nat Commun [8].	Cell model (HUVEC)	Control pLV-IL35 pLV-IL35-sh ICAM1 Fibrinogen pLV-IL-35+fibrinogen pLV-IL-35+fibrinogen+anti-ICAM1 Ab IL-35+anti-GP130 IL-35+anti-IL12R*β*2 IL-35+stat1 inhibitor	Investigate the role of IL-35 in cancer metastasis and progression	IL-35 promotes ICAM1 overexpression through GP130-STAT1 signaling pathway, which facilitates endothelial adhesion and transendothelial migration via an ICAM-fibrinogen-ICAM1 bridge.

Huang et al. 2018 Gastroenterology [19].	PDAC tissues Cell model (monocytes) (HUVEC + monocytes treated accordingly)	pLV-vector pLV-IL35 pLV-scramble pLV-shIL35 pLV-IL35+clophosome Control pLV-IL35 pLV-scramble pLV-shIL35 pLV-IL35+anti-CCL2 pLV-IL35+anti-CCL5 Control Anti-CXCL1 Anti-CXCL8 Anti-CXCL1+ Anti-CXCL8	Investigate the role of IL35 in monocyte-induced angiogenesis of PDAC in mice	IL-35 upregulation in tumor cells recruits monocytes to PDAC tissues via CCL5 while also upregulating angiogenesis-related genes (CXCL1, CXCL8) in monocytes, promoting angiogenesis. IL-35 activates STAT1 and STAT4 phosphorylation through the IL12RB2:gp130 receptor, triggering CCL5 transcription in PDAC and CXCL1, CXCL8 in monocytes.

Zou et al. 2017 Oncotarget [78]	Mice model	Control pUN01 pIL-35	Investigate the effect of IL-35 on neutrophils in tumor development	IL-35 overexpression in neutrophils induced N2 polarization and enhanced their proendothelial angiogenic capabilities.

Liu et al. 2022 FEBS J [88].	Cell model (MB-231, MCF-7) Mice model	Control IL-35 sEVs IL-35-sEVs	Investigate the participation of sEVs derived from breast cancer cells in modulating angiogenesis and the effect of IL-35 in facilitating this process.	IL-35 promoted exosome production in breast cancer cells (IL-35-sEVs), which enhanced HUVEC proliferation and angiogenesis.

*Note:* The references are listed in the order of progression in the text, as shown in the PRISMA flowchart.

Abbreviations: HUVEC, human umbilical vein endothelial cell; ICAM1, intercellular adhesion molecule 1; IL-35, interleukin-35; PDAC, pancreatic ductal adenocarcinoma; sEVs, small extracellular vesicles.

**Table 3:** Studies reporting the effects of IL-35 on endothelial function in rheumatoid arthritis.

Author/year ref.	Type of study	Cohort	Aims	Findings
Jiang et al. 2016 Cell Physiol Biochem [23].	Cell model (HUVEC)	Control VEGF Ang2 VEGF + Ang2 IL-35+VEGF IL-35+Ang2 IL-35+VEGF + Ang2 Anti-Tie2+VEGF Anti-Tie2+Ang2 Anti-Tie2+VEGF + Ang2	Investigate how IL-35 alleviates collagen-induced arthritis	Coculturing RA synovial tissue explants with IL-35 and HUVECs inhibits VEGF-induced HUVEC functions, including wound healing, migration, adhesion, and tube formation, via the Ang2/Tie2 pathway.

Wu et al. 2016 Int Immunopharmacol [24].	Mice model	Control CIA + PBS CIA + IL-35	Investigate the effect of IL-35 on VEGF and its receptors in a CIA mouse model of RA.	IL-35 suppressed the expression of VEGF and its receptors Flt-1 and Flk-1, as well as the inflammatory factor TNF-*α* in synovial tissues

Wu et al. 2018 Clin Exp Rheumatol [25].	Cell model (HUVEC)	CIA + PBS CIA + IL-35 CIA + IL-35+STAT1-inhibitor	Investigate the anti-angiogenic effect of IL-35 in FLS.	IL-35 inhibits the expression of VEGF, FGF-2, endostatin, TNF-*α*, and IL-6 in FLS through STAT1 while promoting the expression of endostatin.

Houshmandi et al. 2016 Cytokine [26].	Mice model	Control IGF-1 IGF-1 antagonist IL-35 IL-35 antagonist IGF-1+IL-35 antagonist	Investigate the mechanism of IL-35 mediating vascular inflammation and endothelial dysfunction via IGF-1	rhIL-35 had no significant effect on inflammatory lesions and neovascularization in arthropathic lesions but inhibited them in lesions with p35 inhibitors.

Liu et al. 2019 Clin Exp Rheumatol [27]	Cell model (MC3T3E1)	Control TNF-*α* TNF-*α*+IL-35	Investigate the function of IL-35 in osteoblast (MC3T3E1) angiogenesis and its signaling pathway in RA	IL-35 promotes MC3T3E1 cell proliferation, upregulates VEGF and its receptors Flt-1 and Flk-1, and inhibits apoptosis under inflammation.

*Note:* The references are listed in the order of progression in the text, as shown in the PRISMA flowchart.

Abbreviations: CIA, collagen-induced arthritis; FLS, fibroblast-like synoviocytes; HUVEC, human umbilical vein endothelial cell; IL-35, interleukin-35; RA, rheumatoid arthritis; VEGF, vascular endothelial growth factor.

## Data Availability

This is a review article, and no new data were generated or analyzed during the preparation of this manuscript. All data referenced in this review were obtained from publicly available sources or published studies, which are cited in the reference list.
